# Risk factors for drug hypersensitivity reactions in children

**DOI:** 10.1186/s13052-024-01694-x

**Published:** 2024-07-15

**Authors:** Francesca Mori, Francesca Saretta, Sara Riscassi, Silvia Caimmi, Paolo Bottau, Lucia Liotti, Fabrizio Franceschini, Annamaria Bianchi, Rocco Luigi Valluzzi, Giuseppe Crisafulli, Carlo Caffarelli

**Affiliations:** 1 Allergy Unit, Meyer Children’s Hospital IRCCS, 50139 Florence, Italy; 2 Dipartimento Materno-Infantile, SC Pediatria, Ospedale Latisana-Palmanova, Azienda Sanitaria Universitaria Friuli Centrale, 33100 Udine, Italy; 3 UOC Di Pediatria, Ospedale Bolzano, Azienda Sanitaria Dell’Alto Adige, Bolzano, 39100 Italy; 4 SC Di Pediatria, Fondazione IRCSS Policlinico San Matteo, 27100 Pavia, Italy; 5 UOC Di Pediatria E Neonatologia, Ospedale Imola (BO), Imola, 40026 Italy; 6 UOC Pediatria, Azienda Ospedaliero-Universitaria “Ospedali Riuniti”, 60100 Ancona, Italy; 7 Private Practice in Allergy, 60100 Ancona, Italy; 8 UOC Pediatria, Azienda Ospedaliera San Camillo Forlanini, 00152 Rome, Italy; 9 Translational Research in Pediatric Specialties Area, Division of Allergy, Bambino Gesù Children’s Hospital, IRCCS, 00165 Rome, Italy; 10 Dipartimento Materno-Infantile, UOC Pediatria, University of Messina, Messina, 98122 Italy; 11 Department of Medicine and Surgery, Clinica Pediatrica, Azienda Ospedaliera-Universitaria, University of Parma, 43123 Parma, Italy

**Keywords:** Drug allergy, Risk factors, Hypersensitivity reactions, Severe cutaneous adverse reaction, Children

## Abstract

Drug hypersensitivity reactions are common in children. Risk factors predisposing to IgE-mediated drug allergies and delayed drug reactions are a matter of debate. Gender, age, previous reactions to the same drug or to another drug, reduced drug metabolism, chronic diseases, polypharmacy, drug doses are linked with the onset of hypersensitivity reactions in some children. Novel advances in genetic polymorphisms can rapidly change the approach to the prevention of reactions since gene testing can be a useful screening test for severe cutaneous adverse reactions. Viral infections may act as cofactors in susceptible individuals. Polypharmacy, high doses, repeated doses and parental route of administration are also risk factors. Clinicians should take into account risk factors to allow the risk–benefit balance to be maintained.

## Introduction

Hypersensitivity reactions (HRs) to drugs are common in the pediatric age. One of the most comprehensive studies suggested that 3% of admissions to a large British children’s hospital were due to adverse drug reactions (ADRs) [1]. However, specific data on risk factors for HRs in children are limited [2]. The knowledge of risk factors for HRs could help in the diagnostic work-up and in preventing new episodes. The risk for HRs in children is due in part to known risk factors and it is in part to the nature of pharmacotherapy [3]. Today seven generally accepted risks for HRs have been found (Table 1) [3]. Risk factors for HRs can be roughly divided into those related to the host and those related to the drug. The aim of this paper is to update and understand risks factors for HRs in children.

**Table 1:** Risk factors for hypersensitivity reactions

Risk Factors	Gender
	Extremes of age
	Previous adverse reactions to the same or to another drug
	Reduced drug metabolism – chronic diseases
	Genetic polymorphisms
	Polypharmacy
	Drug doses

## Risk factors related to the host

### Gender

In a recently published report from the European Anaphylaxis Registry female gender and adulthood were risk factors for drug anaphylaxis [4]. Most studies among adults show that women have a higher incidence of ADRs in general. In pediatric age, conflicting data has been published. The pediatric task force of the EAACI Drug Allergy Interest Group described that female gender has not been associated with increased risk for HRs [5]. It was reported that HRs to non-steroidal anti-inflammatory drugs (NSAIDs) in children, especially in the younger age group, are as frequent if not more frequent in males than in females [6]. In contrast, a prevalence in the female sex has been reported for HRs to omalizumab and to antiepileptic drugs (AEDs). However, unlike adults, it cannot be established that female gender is a risk factor for drug HRs in childhood probably due to differences between adult women and girls in terms of metabolism, hormones, and others still unknown factors [7–9].

### Age

Generally, extreme ages are considered risk factors of HRs [10]. The drug-metabolizing enzymes, the maturation of renal function, the hormone changes over the years may enhance the risk for HRs in children. Regarding AEDs, children are more frequently affected by HRs than adults, in particular the subgroup of those younger than 12 years using aromatic anticonvulsants or multiple AEDs [11]. A recent prospective study examined HRs to AEDs during childhood and revealed that the rate of carbamazepine-associated Drug Reaction with Eosinophilia and Systemic Symptoms (DRESS) was remarkably higher in children than in adults. Moreover, the risk for valproic acid–induced liver injury is higher under five years of age than at other ages. In addition, severe skin rashes due to lamotrigine are more frequent in children under 13 years than in adults [12, 13]. For example, among beta-lactams (BLs), the risk of serum sickness-like reactions to cefaclor is about 1% in toddlers compared to 1:1000 in adults [14]. Several studies on HRs to antibiotics in children also report that children up to 4 years of age more frequently visit drug allergy clinics, suggesting that young children may be more susceptible to antibiotic allergy. Most of these studies are based on parent’s reported clinical history without a drug provocation test proven diagnosis, so such data could probably reflect the high frequency of benign rashes during a viral infection and concomitant drug assumption rather than a true drug HR. An Italian study, in children between 0 and 14 years, showed a higher incidence of ADRs in infants under one year of age with a decreasing incidence with increasing age [15]. About NSAIDs, a quarter of patients with a diagnosed HR are aged 8–18 years [16, 17]. To date, there are few reports on the prevalence of risk factors of HRs to biologics in children. It was only observed an increased risk of HRs to infliximab in younger age [18]. The higher risk of HRs in young children than in older children may be explained also by the fact that in children the risk is increased by off-label prescriptions [5, 7]. On the contrary, it is known that the risk of exposure to many drugs is lower in children than in adults, so it could not explain the increased risk for HRs. Another explanation could be that the widespread prescription of certain drugs in pediatric age, may theoretically increase the risk of immunological sensitization, as drug metabolism is usually age-dependent and an increase in reactive metabolites may occur in young children [7]. Anyway, not all studies are in line with the above reported results and the incidence of HRs by age varies among studies. Other studies found no significant correlation with age or an increased risk of HRs with age [19, 20]. In an international study that aimed to determine the risk factors associated with HR in hospitalized children, subjects older than 11 years showed a significantly higher incidence of HR than those 2–11 years [9]. Faitelson et al. [21] found that older age at the reaction was significantly (*P* = 0.05**)** associated with amoxicillin allergy in children, probably because the exposure to that drug increases with age.

### Atopy

Historically, atopic predisposition has not been accepted as a significant risk factor for drug HRs but it may contribute to more severe allergic reactions [22, 23].

Anyway, the role of atopy and atopic disease in drug HRs is controversial and seems to be related to the type of drug involved in the index reaction. In childhood, atopy, asthma, and chronic urticaria were reported to be significant risk factors for reactions to NSAIDs [24–30]. Allergy to NSAIDs is more common in children with asthma, which is itself a risk factor for more severe reactions to NSAIDs [6]. In recent years, atopy was found to be a risk factor for NSAID hypersensitivity in many studies evaluating adults and children. The influence of atopy on NSAID hypersensitivity seems to vary with the type of reaction [2, 25, 31]. In adults, single NSAID reactors showed a much higher prevalence of atopic diseases than multiple NSAID reactors [32]. In children, hypersensitivity to NSAIDs was significantly associated with personal and/or family atopy, and the relation was especially evident, contrary to adults, in multiple NSAID reactors [33–35].

In line with the above-mentioned results, Alves et al. [36] analyzed a group of 119 pediatric patients (median age nine years) who complained a HR to NSAIDs, being ibuprofen the most frequent culprit drug (79%). After drug provocation test, NSAID hypersensitivity was confirmed in 7.6% children (though inconclusive in 14.3%). Anaphylaxis was a relative risk to NSAID hypersensitivity, while atopy and the number of previous reactions showed no association in single NSAIDs reactors. In children with chronic urticaria, NSAID intake can exacerbate symptoms [37].

Regarding antibiotics, a 20-years study in a large series of children, which evaluated test results and risk factors, found that atopy was not a risk factor for BL allergy [38]. Some other pediatric studies reached similar conclusions, but risk analysis was not performed separately for different drug groups [20, 39, 40]. Arikoglu T et al. [20] found that several risk factors, including atopy (personal or familiar), age and sex, viral infections during index reaction, total IgE or eosinophilia were not related with an increased risk for HRs. Only two studies found a positive correlation between asthma and risk for HRs. Demirhan A et al. [41] demonstrated that, in a group of 204 children investigated for a suspect HR to BLs any atopic disease other than asthma and an interval of reaction of 0–6 h during the index reaction could be considered as risk factors for a true drug HRs. Faitelson Y et al. [21] showed that asthma (*p* = 0.03), and angioedema (*p* = 0.069) were significantly associated with amoxicillin allergy in children with ADRs (*P* = 0.01).

### Family history

It is commonly stated that family history of drug allergy is not a risk factor for HRs, for example maternal penicillin allergy is not a risk factor for drug allergy [42].

Anyway, some other studies showed a positive correlation between family history of drug allergy and risk for HRs. Accordingly, some children with parents who have a true drug allergy are at a 15-fold higher relative risk for allergic reactions to the same drugs [43]. Faitelson Y et al. [21] significantly associated family history of drug allergy with amoxicillin allergy in children with immediate and non-immediate reaction (*p* = 0.01). Arikoglu et al. [20] have evaluated 97 children with 180 suspected drug HRs (mostly antibiotics, NSAIDs and AEDs). In the group of children with a confirmed drug HRs the only two significant risk factors were a strong family history (4.4 times) and a strong personal history of drug allergy (3.5 times). Demirhan et al. [41] have demonstrated that a family history of drug allergy could be considered as risk factors for a true drug HRs. Dias de Castro et al. [19] found a significant association between a positive diagnostic work-up and parental/sibling history of drug allergy (*p* < 0.001), shorter time interval between the suspected reaction and the study (*p* = 0.046) and more severe reactions (*p* < 0.001). A recent study showed an increased risk for positive drug provocation test to BL in children with parental history of drug allergy, but only in non-immediate reactions [44]. Family history of drug allergy was mainly but not exclusively related to BL (66.7%). The limited number of children with confirmed BL allergy did not allow a multivariate analysis and this was a limitation of this study.

### Pharmacogenomics

Class I Human Leucocyte Antigen (HLA) alleles are associated with severe cutaneous drug reactions (SCAR) to certain drugs (Table 2).

**Table 2:** Correlation between HLA patterns, severe cutaneous adverse reactions, and populations

HLA	Type of drug	Drug HR and population	Reference
B*15:02 B*15:21	Carbamazepine	SJS/TEN in Han Chinese in Taiwan, Hong Kong, Thailand, India, but not in Japanese, Europeans of non-Asian ancestry	[45–49]
A*02:01/ Cw*15:02 B*38:01 A*11:01 A*24:02 A*31:01	Phenytoin Phenytoin and lamotrigine Carbamazepine Phenytoin and lamotrigine Carbamazepine	SCAR in Europeans SJS/TEN in Europeans SJS/TEN in Europeans SJS/TEN, DRESS in Europeans DRESS in Europe and Japan	[50]
A*31:01	Carbamazepine	Maculopapular eruption/SJS-TEN/ DRESS in Han Chinese, Northern European, Japanese, and Korean	[45–49]
A*32:01	Vancomicin	DRESS and liver injury in American and Australian with European ancestry	[51]
B*15:02	Phenytoin	SJS in Han Chinese in Hong Kong and Thais, but not with MPE among Han Chinese from Hong Kong	[46, 52]
B*58:01	Allopurinol	SJS/TEN in Han Chinese from Taiwan [OR 580.3], Thais, Japanese, and Europeans	[50, 53–55]
B*57:01	Abacavir	HSR/DRESS in Caucasians [OR 117], but not among blacks. This haplotype has been found to be uncommon in Taiwanese Chinese and Korean populations	[56–58]

Notably, the carriage of a risk allele is not sufficient to identify candidates to develop a HR to the involved drug. So, the value of screening programs based on genotyping in some ethnicity is limited. However, HLA B*57:01 screening has been shown, in a double-blind prospective randomized study, to be useful in preventing HRs to abacavir [59]. On the same line, screening for HLA-B*15:02 before using carbamazepine in Southeast Asian countries [60, 61] and screening for HLA B*58:01 before using allopurinol in Asians [62, 63] prevents HRs to these drugs. The clinical use of genetic markers associated with the risk of HR to other drugs is hampered by the low accuracy due to very high rate of false positive results. Notwithstanding, in some cases, drugs with a known high-risk for severe delayed HRs, as amoxicillin-clavulanate, dapsone, fluocloxacillin, oxcarbazepine, lamotrigine, nevirapine, sulfamethoxazole/trimethoprim, vancomicin have been associated with specific HLA [51, 64–67]. A gene variants of class II alpha chain (HLA-DRA) and the HLA-DRA/HLA-DRB5 interregion have been demonstrated to be a predictor for HRs to penicillins in a genome-wide study in adults [68]. With a genome-wide study, some variants including the HLA-DRB1*07:01 allele, have been associated with an increased risk of HR to asparaginase in children with acute lymphocytic leukemia treated with [69]. HLA-DRB1*16:02 has been associated with PEG-asparaginase hypersensitivity in children [70], confirmed with a univariate and multivariate logistic regression analysis.

### Drug metabolism

In the last ten years the role of “pharmacometabonomic approach” in drug reactions has been evaluated to study drugs’ metabolism, safety, and efficacy in humans. The study of the metabolism of a drug could offer several advantages since it has already been observed some significant associations between some drug HRs and specific drug metabolic profiles. [71, 72] For instance, the metabolizing enzymes cytochrome P450 (CYP) and N-acetyltransferase (NAT) seem to be an important risk factors for certain type of drug HRs such as SCARs [71, 73, 74]. Moreover, some variations in the genes CYP2C9, CYP2C19, and arylamine Nacetyltransferase 2 (NAT2) are associated with a higher risk for drug HR to metamizole [75, 76]. It has been showed that some genetic polymorphisms are associated to anti-tubercolosis (TBC) drug-induced hepatitis [77, 78]. Although no specific pediatric data have been published so far, a few studies included some adolescents too [76, 78]. Kim et al. [79] found a strong association between genetic polymorphisms of CYP and the risk of developing maculo-papular eruption induced by anti-TBC drug in adults.

### Chronic/other disorders

Chronic diseases enhance the risk of DA due to impaired metabolism at kidney and liver [80]. Several studies reported that HRs are more common in children with cystic fibrosis (CF); particularly the frequency of BL HRs is higher. This could be potentially explained by recurrent exposure to drugs, frequent use of intravenous drugs, and specific immune responses related to CF itself [5, 81, 82]. However, the prevalence of BL HRs is lower in children with CF compared to the general pediatric population. Nevertheless, these data require larger prospective studies to be confirmed [5, 83]. Mastocytosis is another reported risk factor for anaphylaxis in children. The frequency of perioperative anaphylaxis appears to be higher in children with mastocytosis compared to the general population [84] therefore the patient should be prepared before general anesthesia to prevent further reactions. It has been highlighted [85] that there were no studies showing a significant increase of specific drug HR in children with mastocytosis, except for perioperative anesthesia. Finally, Kuyucu S et al. [86] reported that HRs to aromatic AEDs in epileptic children are observed more frequently in patients with concomitant other immune system disorders, systemic lupus erythematosus, infectious diseases, and in those who are under treatment with corticosteroids. It has also been shown that the binding of AEDs to proteins is decreased in patients with head trauma, and therefore, head trauma may be a potential risk factor for AED HRs. However, causalities have not been conclusively proven for these risk factors.

### Concomitant infections

Infections often could act as cofactor in drug HRs [87, 88]. Caubet et al. [89] have demonstrated as, even in children with positive oral challenge in healthy conditions, a positivity to a virus was recorded in some cases (mainly Epstein Barr virus (EBV)). The most notable rash is, indeed, the one occurring in patients with EBV infection and treated with aminopenicillin. This peculiar rash has been frequently described since 1960s [90]. Amoxicillin is associated with a 29.5% incidence, but it has been described also for other aminopenicillins [91–93]. A dose dependent widespread altered lymphocyte stimulation is a possible pathomechanism explaining the onset of skin eruption while on antibiotic treatment in patients infected by EBV [94]. Other herpes virus, such a Human herpes virus 6 (HHV6) and cytomegalovirus (CMV) have been associated with similar exanthema. HHV6 has been described in cases of DRESS in association with amoxicillin [95–98]. Mycoplasma Pneumoniae has been associated with a minor rash, as erythema multiforme, but also with SCAR such as Stevens-Johnson Syndrome (SJS) [99–101]. Mycoplasma has been also described as the main cause of a typical rash and mucositis syndrome [102]. Human immunodeficiency virus (HIV) is a frequent cofactor in HR to drugs typically used in this infection, with an incidence from 3 to 20% [103, 104]. HIV infection is also a cofactor in drug HRs to anti-TBC drugs, both in children and adults [105]. Saka et al. [106] have demonstrated, in a mixed population (children and adults) in four sub-Saharan African countries from 2000 to 2010, that sulfonamides were the first cause of drug HRs (38.4%), followed by nevirapine (19.8%) and anti-TBC drugs (5.6%). Importantly, HIV was probably a cofactor also in the severity of reactions. It has also been observed that infants and children seem to develop more drug HRs to trimethoprim-sulfametoxazole compared to adults and even more severe or life-threatening reactions [107–109].

### Previous reactions with the same drug and severity

Moral et al. [110] showed that in a group of 503 patients under 15 years, allergy to BLs was confirmed more frequently in patients with history of repeated or serious reactions to the drugs. These results were confirmed by a more recent study in which patients at risk were considered those with history of multiple reactions (two or more) with the same of different BLs, those with serious reactions, with reactions via parental route and those with immediate urticaria [111]. Some studies reported that immediate reactions have a high probability of being confirmed after a diagnostic work-up [112]. Population specific predicting models and artificial intelligence application are based on historical risk factors such as history of anaphylaxis or multiple episodes [20]. Anyway, a history of recurrent episodes of HRs in young children does not necessarily correlate with a challenge proven diagnosis [113–115]. In fact, at all ages, drug allergy including NSAIDs hypersensitivity is more likely to be confirmed in adults than in children. Children have less exposure to drugs, and maculopapular exanthems due to acute infections are more common [89]. Sipahi Cimen et al. [116] have evaluated 214 children with suspected mild cutaneous reactions to BLs in a 5-years period. BL allergy was diagnosed by oral provocation test in 10.7% of children. A history of proven drug allergy was the only statistically significant risk factor in the multivariate logistic regression analysis.

### Severity of previous reactions

The positive correlation between history of severe reactions and increased risk of BL allergy was reported by Ponvert C et al. [38]. On the other hand, several risk factors (history of asthma, food allergy, multiple operations, family history of atopy) [117], seem to increase the probability of severe reactions in perioperative anaphylactic reactions. This conclusion is not confirmed by other pediatric studies where the demographic features including age and sex, frequency of atopy, allergic diseases, chronic diseases, and family history of allergic diseases were similar between the patients with and without anaphylaxis, as well as between the drug hypersensitive patients and the participants without any history of drug HRs [118]. Prior data demonstrate that patients who report an anaphylactic history have a 2- to fourfold increased risk of true allergy [119]. Anaphylactic history additionally confers an increased risk of anaphylaxis during allergy testing [22], and cross-reactivity with other BLs [120]. Furthermore, a recent study of 182 patients with positive challenge tests to BLs showed that the only clinical risk factor for developing anaphylaxis during drug challenge was an index reaction of anaphylaxis, with more than a tenfold risk increase observed [121]. The severity and the time latency of the reactions seem to be both important in confirming drug hypersensitivity. A higher risk of penicillin allergy was associated with severe delayed reactions (at any point in the past) and severe immediate symptoms (in particular if occurred within 5 years from the event) [122].

## Risk Factors related to the drug

Drug-related factors, including molecular weight, polypharmacy, frequency of exposure, and route of administration, are reported as additional risk factors for HRs. Immunogenicity of drugs is affected by include its ability to act as a hapten, a prohapten or to bind covalently to immune receptors (Pi concept). Thus, certain classes of drugs tend to induce a higher rate of drug HR compared with others. This is the case of large macromolecular drugs (e.g., insulin or horse antisera) or drugs that haptenize (bind to tissue or blood proteins and elicit an immune response), such as penicillin, that are also associated with a greater likelihood of causing HRs [123].

### Multiple therapies and doses

The risk of reaction increases with the number of drugs taken [43] and polypharmacy is considered a constant risk factor of HRs [5, 124–126]. Exposure to multiple drugs may reduce the threshold at which patients demonstrate allergic responses [127]. In the study by Guvenir H et al. [11] children who used a single AED had a lower frequency of HRs than those who used multiple AEDs, 3.8% *vs* 4.4%, respectively. The same results have been observed by Lalic M et al. [128] who additionally demonstrated that high starting dose AEDs was another risk factor. Moreover, prolonged high dose repeated exposures after treatment suspension, are more likely to lead to HRs than a large single dose since they enhance immune recognition [80, 129]. Finally, children are at special risks for HRs since unfriendly dosing formulations of drugs or miscalculation of some health care professional can lead to tenfold overdoses in administration of medicine. Although children are less likely to be repeatedly exposed to drugs that is necessary for sensitization to occur, widespread prescription of certain drugs may theoretically increase the risk of sensitization in certain groups of children, for instance antibiotic sensitization in children with chronic diseases including chronic infections requiring multiple drugs therapies (i.e. HIV infections). Anyway, most of immediate reactions occurred on first exposure without a pre-sensitizing phase required, while delayed reactions were more common on regimen with frequent interval [130].

### Route of administration

Topical, intramuscular, and intravenous routes of administration are more likely to cause allergic drug reactions than oral administration. Moreover, parental administration is associated with more severe reactions because of a more rapid absorption and presentation to immune system [80, 129, 131].

## Conclusions

So far, focused papers on risk factors for HRs in children are lacking, anyway in Tables 3 and 4 risk factors related to the drugs and to the host have been summarized, respectively. A consensus has been reached on the predisposing role of some factors, especially on that of family history and gender. A previous reaction to the same or a related drug is the most significant one. History of anaphylactic shock, multiple reactions, symptoms occurring within 1 h, family history of drug allergy have been reported to be the most important variables for prediction of HRs to BLs [19, 38, 117, 120, 132, 133]. Generally, young adults are more likely to react than infants to certain drugs (i.e. NSAIDs). Although it is believed that parental routes of administration, and prolonged or intermittent and repeated dosing regimens (e.g., for CF) [134] appear to be more sensitizing than a continuous treatment, or the oral route, high-quality studies on this topic are lacking. Polypharmacy is a common risk factor and many of these children have impairment in liver or kidney function. Moreover, underlying viral infections, such as HHV 6, HIV and EBV, can lead to skin eruptions and mimic allergic reactions if a drug (mostly an antibiotic) is taken simultaneously, or may act as cofactors in susceptible individuals [5, 89]. When making treatment decisions, clinicians need to maintain the risk–benefit balance. So, they should know the very real likelihood of HRs since most children do not have a recognizable risk factor. Therefore, more studies are needed to create predictive models to prevent severe HRs.

**Table 3:** Risk factors related to the drugs

Related to the drugs	Increased risk	References
*Polypharmacy*	Single AED 3.8% vs Multiple AED 4.4%	11
*Route of administration*	Topical, intramuscular, intravenoues route	81, 130, 132
*Molecular structures*	Large macromolecules (insulin) or with tendency to haptenize (penicillin)	124

**Table 4:** Risk factors related to the host

Related to the host	Increased risk	References
*Gender*	No gender associaton	5, 7–9
*Age*	AEDs in children younger 12 years VPA related liver injury in children younger 5 years Lamotrigine related SCAR in children younger 13 years	11–13
	Antibiotics Cefaclor related SSLR in toddlers Amoxicillin in older age	14, 15, 21
	NSAIDs in children 8–18 years	16, 17
	Infliximab in younger age	18
*Atopy*	NSAIDs in children with atopy, asthma, chronic urticaria	24–30
*Family history*	Amoxicillin in children with family history	21
	Antibiotics, NSAIDs and AEDs in children with strong family history	20
*Pharmacogenomics*	Several specific HLA patterns	
*Chronic/other diseases*	Children with impaired liver and kidney functions	81
	Children with CF, especially for BLs	5, 82, 83
	Mastocytosis, especially for perioperative drugs	86
	AEDs in children with epilepsy and other associated immune system disorders	87
*Concomitant infections*	Viral infections	90
	Amoxicillin/aminopenicillins in children with EBV infections	91–95
	Amoxicillin in children with DRESS and HHV6 infections	96–99
	SCARs in children with Mycoplasma Pn infections	100–103
	HIV	104–106
*Previous reactions*	Repeated or serious reaction with the same drug	111
	Proven reactions to BLs	117
*Severe reactions*	BLs and severe reactions	39
	BLs in children wiith a index reactions of anaphylaxis (during drug challenges)	122

## Data Availability

Data sharing is not applicable to this article as no datasets were generated or analysed during the current study.

## Abbreviations

ADR: Adverse drug reaction
AED: Antiepileptic drug
BL: Beta-lactam
CF: Cystic Fibrosis
CMV: Cytomegalovirus
CYP: Cytochrome P450
EBV: Epstein-Barr virus
DRESS: Drug Reaction with Eosinophilia and Systemic Symptoms
HHV6: Human herpes virus 6
HIV: Human immunodeficiency virus
HLA: Human Leucocyte Antigen
HR: Hypersensitivity reaction
NAT: N-acetyltransferase
NAT2: Arylamine Nacetyltransferase 2
NSAID: Non-steroidal anti-inflammatory drug
SCAR: Severe cutaneous adverse reactions
SJS: Stevens-Johnson Syndrome
TBC: Tuberculosis

## References

[1] Gallagher RM, Bird KA, Mason JR, Peak M, Williamson PR, Nunn AJ (2011). Adverse drug reactions causing admission to a paediatric hospital: a pilot study. J Clin Pharm Ther.

[2] Cousin M, Chiriac A, Molinari N, Demoly P, Caimmi D (2016). Phenotypical characterization of children with hypersensitivity reactions to NSAIDs. Pediatr Allergy Immunol.

[3] Rieder M (2012). New ways to detect adverse drug reactions in pediatrics. Pediatr Clin North Am.

[4] Hanschmann T, Francuzik W, Dölle-Bierke S, Hofmeier KS, Grabenhenrich L, Ruëff F (2023). Different phenotypes of drug-induced anaphylaxis-Data from the European Anaphylaxis Registry. Allergy.

[5] Gomes ER, Brockow K, Kuyucu S, Saretta F, Mori F, Blanca-Lopez N (2016). Drug hypersensitivity in children: report from the pediatric task force of the EAACI Drug Allergy Interest Group. Allergy.

[6] Kidon MI, See Y (2004). Adverse drug reactions in Singaporean children. Singapore Med J.

[7] Rebelo Gomes E, Geraldes L, Gaspar Â, Malheiro D, Cadinha S, Abreu C (2016). Hypersensitivity reactions to nonsteroidal anti-inflammatory drugs among adults: Clinical features and risk factors for diagnosis confirmation. Int Arch Allergy Immunol.

[8] Stepanovic B, Sommerfield D, Lucas M, von Ungern-Sternberg BS (2019). An update on allergy and anaphylaxis in pediatric anesthesia. Paediatr Anaesth.

[9] Park JS, Suh DI (2020). Drug allergy in children: what should we know?. Clin Exp Pediatr.

[10] Rieder M (2019). Adverse Drug Reactions in Children: Pediatric Pharmacy and Drug Safety. J Pediatr Pharmacol Ther.

[11] Guvenir H, Dibek Misirlioglu E, Civelek E, Toyran M, Buyuktiryaki B, Ginis T (2018). The frequency and clinical features of hypersensitivity reactions to antiepileptic drugs in children: A prospective study. J Allergy Clin Immunol Pract.

[12] Anderson GD (2002). Children versus adults: pharmacokinetic and adverse-effect differences. Epilepsia.

[13] Hirsch LJ, Weintraub DB, Buchsbaum R, Spencer HT, Straka T, Hager M (2006). Predictors of Lamotrigine-associated rash. Epilepsia.

[14] Kearns GL, Wheeler JG, Rieder MJ, Reid J (1998). Serum sickness-like reaction to cefaclor: lack of in vitro cross-reactivity with loracarbef. Clin Pharmacol Ther.

[15] Menniti-Ippolito G, Raschetti R, Da Cas R, Giaquinto C, Cantarutti L (2000). Active monitoring of adverse drug reactions in children. Italian Paediatric Pharmacosurveillance Multicenter Group Lancet.

[16] Sánchez-Borges M, Capriles-Behrens E, Caballero-Fonseca F (2004). Hypersensitivity to non-steroidal anti-inflammatory drugs in childhood. Pediatr Allergy Immunol.

[17] Kidon M, Blanca-Lopez N, Gomes E, Terreehorst I, Tanno L, Ponvert C (2018). EAACI/ENDA Position Paper: Diagnosis and management of hypersensitivity reactions to non-steroidal anti-inflammatory drugs (NSAIDs) in children and adolescents. Pediatr Allergy Immunol.

[18] Mori F, Saretta F, Bianchi A, Crisafulli G, Caimmi S, Liotti L (2020). Hypersensitivity Reactions to Monoclonal Antibodies in Children. Medicina (Kaunas).

[19] Dias de Castro E, Carolino F, Carneiro-Leão L, Barbosa J, Ribeiro L, Cernadas JR. Allergy to beta-lactam antibiotics in children: Risk factors for a positive diagnostic work-up. Allergol Immunopathol (Madr). 2020;48:417–23. 10.1016/j.aller.2020.01.005 32460994

[20] Arikoglu T, Aslan G, Batmaz SB, Eskandari G, Helvaci I, Kuyucu S (2015). Diagnostic evaluation and risk factors for drug allergies in children: from clinical history to skin and challenge tests. Int J Clin Pharm.

[21] Faitelson Y, Boaz M, Dalal I (2018). Asthma, Family History of Drug Allergy, and Age Predict Amoxicillin Allergy in Children. J Allergy Clin Immunol Pract.

[22] Co Minh H-B, Bousquet PJ, Fontaine C, Kvedariene V, Demoly P (2006). Systemic reactions during skin tests with beta-lactams: a risk factor analysis. J Allergy Clin Immunol.

[23] Joint Task Force on Practice Parameters, American Academy of Allergy, Asthma and Immunology, American College of Allergy, Asthma and Immunology, Joint Council of Allergy, Asthma and Immunology. Drug allergy: an updated practice parameter. Ann Allergy Asthma Immunol. 2010;105:259–73. 10.1016/j.anai.2010.08.002 20934625

[24] Sánchez-Borges M, Capriles-Hulett A (2000). Atopy is a risk factor for non-steroidal anti-inflammatory drug sensitivity. Ann Allergy Asthma Immunol.

[25] Hassani A, Ponvert C, Karila C, Le Bourgeois M, De Blic J, Scheinmann P (2008). Hypersensitivity to cyclooxygenase inhibitory drugs in children: a study of 164 cases. Eur J Dermatol.

[26] Quiralte J, Blanco C, Delgado J, Ortega N, Alcntára M, Castillo R (2007). Challenge-based clinical patterns of 223 Spanish patients with nonsteroidal anti-inflammatory-drug-induced-reactions. J Investig Allergol Clin Immunol.

[27] Ponvert C (2007). Scheinmann P [Allergic and pseudoallergic reactions to analgesics, antipyretics and non-steroidal anti-inflammatory drugs]. Arch Pediatr.

[28] Kidon MI, Kang LW, Chin CW, Hoon LS, See Y, Goh A (2005). Early presentation with angioedema and urticaria in cross-reactive hypersensitivity to nonsteroidal antiinflammatory drugs among young, Asian, atopic children. Pediatrics.

[29] Ayuso P, Blanca-López N, Doña I, Torres MJ, Guéant-Rodríguez RM, Canto G (2013). Advanced phenotyping in hypersensitivity drug reactions to NSAIDs. Clin Exp Allergy.

[30] Lo P-C, Tsai Y-T, Lin S-K, Lai J-N (2016). Risk of asthma exacerbation associated with nonsteroidal anti-inflammatory drugs in childhood asthma: A nationwide population-based cohort study in Taiwan. Medicine (Baltimore).

[31] Sánchez-Borges M, Caballero-Fonseca F, Capriles-Hulett A (2016). Cofactors and comorbidities in patients with aspirin/NSAID hypersensitivity. Allergol Immunopathol (Madr).

[32] Asero R (2015). Single NSAID hypersensitivity is associated with atopic status. Eur Ann Allergy Clin Immunol.

[33] Blanca-López N, Cornejo-García JA, Plaza-Serón MC, Doña I, Torres-Jaén MJ, Canto G (2015). Hypersensitivity to Nonsteroidal Anti-inflammatory Drugs in Children and Adolescents: Cross-Intolerance Reactions. J Investig Allergol Clin Immunol.

[34] Zambonino MA, Torres MJ, Muñoz C, Requena G, Mayorga C, Posadas T (2013). Drug provocation tests in the diagnosis of hypersensitivity reactions to non-steroidal anti-inflammatory drugs in children. Pediatr Allergy Immunol.

[35] Arikoglu T, Aslan G, Yildirim DD, Batmaz SB, Kuyucu S (2016). Discrepancies in the diagnosis and classification of nonsteroidal anti-inflammatory drug hypersensitivity reactions in children. Allergol Int.

[36] Alves C, Romeira AM, Abreu C, Carreiro-Martins P, Gomes E, Leiria-Pinto P (2017). Non-steroidal anti-inflammatory drug hypersensitivity in children. Allergol Immunopathol (Madr).

[37] Tsabouri S, Arasi S, Beken B, Church MK, Alvaro-Lozano M, Caffarelli C (2022). A European survey of management approaches in chronic urticaria in children: EAACI pediatric urticaria taskforce. Pediatr Allergy Immunol.

[38] Ponvert C, Perrin Y, Bados-Albiero A, Le Bourgeois M, Karila C, Delacourt C (2011). Allergy to betalactam antibiotics in children: results of a 20-year study based on clinical history, skin and challenge tests. Pediatr Allergy Immunol.

[39] Tugcu GD, Cavkaytar O, Sekerel BE, Sackesen C, Kalayci O, Tuncer A (2015). Actual drug allergy during childhood: Five years’ experience at a tertiary referral centre. Allergol Immunopathol (Madr).

[40] Indradat S, Veskitkul J, Pacharn P, Jirapongsananuruk O, Visitsunthorn N (2016). Provocation proven drug allergy in Thai children with adverse drug reactions. Asian Pac J Allergy Immunol.

[41] Demirhan A, Yildirim DD, Arikoglu T, Ozhan AK, Tokmeci N, Yuksek BC (2021). A combined risk modeling strategy for clinical prediction of beta-lactam allergies in children. Allergy Asthma Proc.

[42] Wu P, Longbottom K, Hague R, Vance G (2018). Fifteen-minute consultation: A child with a suspected drug allergy. Arch Dis Child Educ Pract Ed.

[43] Sharma VK, Dhar S (1995). Clinical pattern of cutaneous drug eruption among children and adolescents in north India. Pediatr Dermatol.

[44] Mill C, Primeau M-N, Medoff E, Lejtenyi C, O’Keefe A, Netchiporouk E (2016). Assessing the Diagnostic Properties of a Graded Oral Provocation Challenge for the Diagnosis of Immediate and Nonimmediate Reactions to Amoxicillin in Children. JAMA Pediatr.

[45] Chung W-H, Hung S-I, Hong H-S, Hsih M-S, Yang L-C, Ho H-C (2004). Medical genetics: a marker for Stevens-Johnson syndrome. Nature.

[46] Locharernkul C, Loplumlert J, Limotai C, Korkij W, Desudchit T, Tongkobpetch S (2008). Carbamazepine and phenytoin induced Stevens-Johnson syndrome is associated with HLA-B*1502 allele in Thai population. Epilepsia.

[47] Mehta TY, Prajapati LM, Mittal B, Joshi CG, Sheth JJ, Patel DB (2009). Association of HLA-B*1502 allele and carbamazepine-induced Stevens-Johnson syndrome among Indians. Indian J Dermatol Venereol Leprol.

[48] Kaniwa N, Saito Y, Aihara M, Matsunaga K, Tohkin M, Kurose K (2008). HLA-B locus in Japanese patients with anti-epileptics and allopurinol-related Stevens-Johnson syndrome and toxic epidermal necrolysis. Pharmacogenomics.

[49] McCormack M, Alfirevic A, Bourgeois S, Farrell JJ, Kasperavičiūtė D, Carrington M (2011). HLA-A*3101 and carbamazepine-induced hypersensitivity reactions in Europeans. N Engl J Med.

[50] Ramírez E, Bellón T, Tong HY, Borobia AM, de Abajo FJ, Lerma V (2017). Significant HLA class I type associations with aromatic antiepileptic drug (AED)-induced SJS/TEN are different from those found for the same AED-induced DRESS in the Spanish population. Pharmacol Res.

[51] Asif BA, Koh C, Phillips EJ, Gu J, Li YJ, Barnhart H (2024). Vancomycin-induced liver injury, DRESS, and HLA-A*32:01. J Allergy Clin Immunol Pract.

[52] Man CBL, Kwan P, Baum L, Yu E, Lau KM, Cheng ASH (2007). Association between HLA-B*1502 allele and antiepileptic drug-induced cutaneous reactions in Han Chinese. Epilepsia.

[53] Hung S-I, Chung W-H, Liou L-B, Chu C-C, Lin M, Huang H-P (2005). HLA-B*5801 allele as a genetic marker for severe cutaneous adverse reactions caused by allopurinol. Proc Natl Acad Sci U S A.

[54] Lonjou C, Thomas L, Borot N, Ledger N, de Toma C, LeLouet H (2006). A marker for Stevens-Johnson syndrome …: ethnicity matters. Pharmacogenomics J.

[55] Tassaneeyakul W, Jantararoungtong T, Chen P, Lin P-Y, Tiamkao S, Khunarkornsiri U (2009). Strong association between HLA-B*5801 and allopurinol-induced Stevens-Johnson syndrome and toxic epidermal necrolysis in a Thai population. Pharmacogenet Genomics.

[56] Mallal S, Nolan D, Witt C, Masel G, Martin AM, Moore C (2002). Association between presence of HLA-B*5701, HLA-DR7, and HLA-DQ3 and hypersensitivity to HIV-1 reverse-transcriptase inhibitor abacavir. Lancet.

[57] Hughes AR, Mosteller M, Bansal AT, Davies K, Haneline SA, Lai EH (2004). Association of genetic variations in HLA-B region with hypersensitivity to abacavir in some, but not all, populations. Pharmacogenomics.

[58] Park WB, Choe PG, Song K-H, Lee S, Jang H-C, Jeon JH (2009). Should HLA-B*5701 screening be performed in every ethnic group before starting abacavir?. Clin Infect Dis.

[59] Mallal S, Phillips E, Carosi G, Molina J-M, Workman C, Tomazic J (2008). HLA-B*5701 screening for hypersensitivity to abacavir. N Engl J Med.

[60] Amstutz U, Shear NH, Rieder MJ, Hwang S, Fung V, Nakamura H (2014). Recommendations for HLA-B*15:02 and HLA-A*31:01 genetic testing to reduce the risk of carbamazepine-induced hypersensitivity reactions. Epilepsia.

[61] Ferrell PB, McLeod HL (2008). Carbamazepine, HLA-B*1502 and risk of Stevens-Johnson syndrome and toxic epidermal necrolysis: US FDA recommendations. Pharmacogenomics.

[62] Nuki G (2014). An appraisal of the 2012 American College of Rheumatology Guidelines for the Management of Gout. Curr Opin Rheumatol.

[63] Saokaew S, Tassaneeyakul W, Maenthaisong R, Chaiyakunapruk N (2014). Cost-effectiveness analysis of HLA-B*5801 testing in preventing allopurinol-induced SJS/TEN in Thai population. PLoS ONE.

[64] Khan DA, Banerji A, Blumenthal KG, Phillips EJ, Solensky R, White AA (2022). Drug allergy: A 2022 practice parameter update. J Allergy Clin Immunol.

[65] Cornejo-Garcia JA, Oussalah A, Blanca M, Gueant-Rodriguez R-M, Mayorga C, Waton J (2016). Genetic Predictors of Drug Hypersensitivity. Curr Pharm Des.

[66] Karlin E, Phillips E (2014). Genotyping for severe drug hypersensitivity. Curr Allergy Asthma Rep.

[67] Urban TJ, Daly AK, Aithal GP (2014). Genetic basis of drug-induced liver injury: present and future. Semin Liver Dis.

[68] Guéant J-L, Romano A, Cornejo-Garcia J-A, Oussalah A, Chery C, Blanca-López N (2015). HLA-DRA variants predict penicillin allergy in genome-wide fine-mapping genotyping. J Allergy Clin Immunol.

[69] Fernandez CA, Smith C, Yang W, Mullighan CG, Qu C, Larsen E (2015). Genome-wide analysis links NFATC2 with asparaginase hypersensitivity. Blood.

[70] Liu S, Gao C, Wu Y, Lin W, Li J, Xue T (2021). HLA-DRB1*16:02 is associated with PEG-asparaginase hypersensitivity. Pharmacogenomics.

[71] Agúndez JAG, Mayorga C, García-Martin E (2015). Drug metabolism and hypersensitivity reactions to drugs. Curr Opin Allergy Clin Immunol.

[72] Lee A-Y, Kim M-J, Chey W-Y, Choi J, Kim B-G (2004). Genetic polymorphism of cytochrome P450 2C9 in diphenylhydantoin-induced cutaneous adverse drug reactions. Eur J Clin Pharmacol.

[73] Clayton TA, Lindon JC, Cloarec O, Antti H, Charuel C, Hanton G (2006). Pharmaco-metabonomic phenotyping and personalized drug treatment. Nature.

[74] Lavergne SN, Park BK, Naisbitt DJ (2008). The roles of drug metabolism in the pathogenesis of T-cell-mediated drug hypersensitivity. Curr Opin Allergy Clin Immunol.

[75] Martínez C, Andreu I, Amo G, Miranda MA, Esguevillas G, Torres MJ (2014). Gender and functional CYP2C and NAT2 polymorphisms determine the metabolic profile of metamizole. Biochem Pharmacol.

[76] García-Martín E, Esguevillas G, Blanca-López N, García-Menaya J, Blanca M, Amo G (2015). Genetic determinants of metamizole metabolism modify the risk of developing anaphylaxis. Pharmacogenet Genomics.

[77] Huang Y-S, Chern H-D, Su W-J, Wu J-C, Chang S-C, Chiang C-H (2003). Cytochrome P450 2E1 genotype and the susceptibility to antituberculosis drug-induced hepatitis. Hepatology.

[78] Kim S-H, Kim S-H, Bahn J-W, Kim Y-K, Chang Y-S, Shin E-S (2009). Genetic polymorphisms of drug-metabolizing enzymes and anti-TB drug-induced hepatitis. Pharmacogenomics.

[79] Kim S-H, Kim S-H, Yoon HJ, Shin DH, Park SS, Kim Y-S (2011). NAT2, CYP2C9, CYP2C19, and CYP2E1 genetic polymorphisms in anti-TB drug-induced maculopapular eruption. Eur J Clin Pharmacol.

[80] Doña I, Torres MJ, Celik G, Phillips E, Tanno LK, Castells M (2024). Changing patterns in the epidemiology of drug allergy. Allergy.

[81] Whitaker P, Naisbitt D, Peckham D (2012). Nonimmediate β-lactam reactions in patients with cystic fibrosis. Curr Opin Allergy Clin Immunol.

[82] Caimmi S, Sanfiorenzo C, Caimmi D, Bousquet P-J, Chiron R, Demoly P (2012). Comprehensive allergy work-up is mandatory in cystic fibrosis patients who report a history suggestive of drug allergy to beta-lactam antibiotics. Clin Transl Allergy.

[83] Matar R, Le Bourgeois M, Scheinmann P, de Blic J, Ponvert C (2014). Beta-lactam hypersensitivity in children with cystic fibrosis: a study in a specialized pediatric center for cystic fibrosis and drug allergy. Pediatr Allergy Immunol.

[84] Atanaskovic-Markovic M, Gomes E, Cernadas JR, du Toit G, Kidon M, Kuyucu S (2019). Diagnosis and management of drug-induced anaphylaxis in children: An EAACI position paper. Pediatr Allergy Immunol.

[85] Mori F, Crisafulli G, Bianchi A, Bottau P, Caimmi S, Franceschini F (2022). Drugs and vaccines hypersensitivity in children with mastocytosis. J Clin Med.

[86] Kuyucu S, Caubet J-C (2018). Hypersensitivity reactions to antiepileptic drugs in children: Epidemiologic, pathogenetic, clinical, and diagnostic aspects. J Allergy Clin Immunol Pract.

[87] Shiohara T, Kano Y (2007). A complex interaction between drug allergy and viral infection. Clin Rev Allergy Immunol.

[88] Adam J, Pichler WJ, Yerly D (2011). Delayed drug hypersensitivity: models of T-cell stimulation. Br J Clin Pharmacol.

[89] Caubet J-C, Kaiser L, Lemaître B, Fellay B, Gervaix A, Eigenmann PA (2011). The role of penicillin in benign skin rashes in childhood: a prospective study based on drug rechallenge. J Allergy Clin Immunol.

[90] Patel BM (1967). Skin rash with infectious mononucleosis and ampicillin. Pediatrics.

[91] Thompson DF, Ramos CL (2017). Antibiotic-induced rash in patients with infectious mononucleosis. Ann Pharmacother.

[92] Chovel-Sella A, Ben Tov A, Lahav E, Mor O, Rudich H, Paret G (2013). Incidence of rash after amoxicillin treatment in children with infectious mononucleosis. Pediatrics.

[93] Jappe U (2007). Amoxicillin-induced exanthema in patients with infectious mononucleosis: allergy or transient immunostimulation?. Allergy.

[94] Ónodi-Nagy K, Kinyó Á, Meszes A, Garaczi E, Kemény L, Bata-Csörgő Z (2015). Amoxicillin rash in patients with infectious mononucleosis: evidence of true drug sensitization. Allergy Asthma Clin Immunol.

[95] Chen Y-C, Chiang H-H, Cho Y-T, Chang C-Y, Chen K-L, Yang C-W (2015). Human herpes virus reactivations and dynamic cytokine profiles in patients with cutaneous adverse drug reactions –a prospective comparative study. Allergy.

[96] Ozcan D, Seçkin D, Bilezikçi B, Arslan H (2010). The role of human herpesvirus-6, Epstein-Barr virus and cytomegalovirus infections in the etiopathogenesis of different types of cutaneous drug reactions. Int J Dermatol.

[97] Mori F, Caffarelli C, Caimmi S, Bottau P, Liotti L, Franceschini F (2019). Drug reaction with eosinophilia and systemic symptoms (DRESS) in children. Acta Biomed.

[98] Hubiche T, Milpied B, Cazeau C, Taïeb A, Léauté-Labrèze C (2011). Association of immunologically confirmed delayed drug reaction and human herpesvirus 6 viremia in a pediatric case of drug-induced hypersensitivity syndrome. Dermatology.

[99] Ravin KA, Rappaport LD, Zuckerbraun NS, Wadowsky RM, Wald ER, Michaels MM (2007). Mycoplasma pneumoniae and atypical Stevens-Johnson syndrome: a case series. Pediatrics.

[100] Ahluwalia J, Wan J, Lee DH, Treat J, Yan AC (2014). Mycoplasma-associated Stevens-Johnson syndrome in children: retrospective review of patients managed with or without intravenous immunoglobulin, systemic corticosteroids, or a combination of therapies. Pediatr Dermatol.

[101] Liotti L, Caimmi S, Bottau P, Bernardini R, Cardinale F, Saretta F, Mori F, Crisafulli G, Franceschini F, Caffarelli C (2019). Clinical features, outcomes and treatment in children with drug induced Stevens-Johnson syndrome and toxic epidermal necrolysis. Acta Biomed.

[102] Lofgren D, Lenkeit C (2021). Mycoplasma Pneumoniae-induced rash and mucositis: A systematic review of the literature. Spartan Med Res J.

[103] Phillips E, Mallal S (2007). Drug hypersensitivity in HIV. Curr Opin Allergy Clin Immunol.

[104] Knight L, Muloiwa R, Dlamini S, Lehloenya RJ (2014). Factors associated with increased mortality in a predominantly HIV-infected population with Stevens Johnson syndrome and toxic epidermal necrolysis. PLoS ONE.

[105] Breen RA, Miller RF, Gorsuch T (2006). Adverse events and treatment interruption in tuberculosis patients with and without HIV co-infection. Thorax.

[106] Saka B, Barro-Traoré F, Atadokpédé FA, Kobangue L, Niamba PA, Adégbidi H (2013). Stevens-Johnson syndrome and toxic epidermal necrolysis in sub-Saharan Africa: a multicentric study in four countries. Int J Dermatol.

[107] Adegbidi H, Atadokpede F, Sagbo G (2012). Drug eruptions in children in Cotonou. Benin Mali Med.

[108] Coopman SA, Johnson RA, Platt R, Stern RS (1993). Cutaneous disease and drug reactions in HIV infection. N Engl J Med.

[109] Chanock SJ, Luginbuhl LM, McIntosh K, Lipshultz SE (1994). Life-threatening reaction to trimethoprim/sulfamethoxazole in pediatric human immunodeficiency virus infection. Pediatrics.

[110] Moral L, Roig M, Toral T, Fuentes MJ, Martos MD, Garde J (2008). Alergia a antibióticos betalactámicos en una población pediátrica: descripción de una serie amplia. Allergol Immunopathol.

[111] Moral L, Garde J, Toral T, Fuentes MJ, Marco N (2011). Short protocol for the study of paediatric patients with suspected betalactam antibiotic hypersensitivity and low risk criteria. Allergol Immunopathol (Madr).

[112] Zambonino MA, Corzo JL, Munoz C, Requena G, Ariza A, Mayorga C (2014). Diagnostic evaluation of hyper- sensitivity reactions to beta-lactam antibiotics in a large population of children. Pediatr Allergy Immunol.

[113] Blanca-Lopez N, Cornejo JA, Perez-Sanchez NI (2017). Hypersensitivity reactions to NSAIDs in children. J Allergy Clin Immunol..

[114] Topal E, Celiksoy MH, Catal F, Gamze Sayan Y, Sancak R (2016). The value of the clinical history for the diagnosis of immediate nonsteroidal anti-inflammatory drug hypersensitivity and safe alternative drugs in children. Allergy Asthma Proc.

[115] Caffarelli C, Franceschini F, Caimmi D, Mori F, Diaferio L, Di Mauro D (2018). SIAIP position paper: provocation challenge to antibiotics and non-steroidal anti-inflammatory drugs in children. Ital J Pediatr.

[116] Sipahi Cimen S, Hizli Demirkale Z, Yucel E, Ozceker D, Suleyman A, Sayili U (2023). Risk factors of challenge-proven beta-lactam allergy in children with immediate and non-immediate mild cutaneous reactions. Int Arch Allergy Immunol.

[117] Vorobeichik L, Weber EA, Tarshis J (2018). Misconceptions surrounding penicillin allergy: Implications for anesthesiologists. Anesth Analg.

[118] Cavkaytar O, Karaatmaca B, Arik Yilmaz E, Sahiner UM, Sackesen C, Sekerel BE (2016). Basal serum tryptase is not a risk factor for immediate-type drug hypersensitivity during childhood. Pediatr Allergy Immunol.

[119] Chiriac AM, Wang Y, Schrijvers R, Bousquet PJ, Mura T, Molinari N (2018). Designing predictive models for beta-lactam allergy using the drug allergy and hypersensitivity database. J Allergy Clin Immunol Pract.

[120] Romano A, Valluzzi RL, Caruso C, Maggioletti M, Quaratino D, Gaeta F (2018). Cross-Reactivity and Tolerability of Cephalosporins in Patients with IgE-Mediated Hypersensitivity to Penicillins. J Allergy Clin Immunol Pract.

[121] Chiriac A-M, Rerkpattanapipat T, Bousquet P-J, Molinari N, Demoly P (2017). Optimal step doses for drug provocation tests to prove beta-lactam hypersensitivity. Allergy.

[122] Stone CA, Trubiano J, Coleman DT, Rukasin CRF, Phillips EJ (2020). The challenge of de-labeling penicillin allergy. Allergy.

[123] Warrington R, Silviu-Dan F, Wong T (2018). Drug allergy. Allergy Asthma. Clin Immunol.

[124] Impicciatore P, Choonara I, Clarkson A, Provasi D, Pandolfini C, Bonati M (2001). Incidence of adverse drug reactions in paediatric in/out-patients: a systematic review and meta-analysis of prospective studies. Br J Clin Pharmacol.

[125] Gallagher RM, Mason JR, Bird KA, Kirkham JJ, Peak M, Williamson PR (2012). Adverse drug reactions causing admission to a paediatric hospital. PLoS ONE.

[126] Rashed AN, Wong ICK, Cranswick N, Tomlin S, Rascher W, Neubert A (2012). Risk factors associated with adverse drug reactions in hospitalised children: international multicentre study. Eur J Clin Pharmacol.

[127] Summers CW, Pumphrey RS, Woods CN, McDowell G, Pemberton PW, Arkwright PD (2008). Factors predicting anaphylaxis to peanuts and tree nuts in patients referred to a specialist center. J Allergy Clin Immunol.

[128] Lalic M, Cvejic J, Popovic J, Bozic K, Golocorbin-Kon S, Al-Salami H (2009). Lamotrigine and valproate pharmacokinetics interactions in epileptic patients. Eur J Drug Metab Pharmacokinet.

[129] Choed-Amphai C, Khorana J, Sathitsamitphong L, Natesirinilkul R, Charoenkwan P (2024). Predictive factors for L-asparaginase hypersensitivity in pediatric acute lymphoblastic leukemia. Int J Hematol.

[130] Kugathasan S, Levy MB, Saeian K, Vasilopoulos S, Kim JP, Prajapati D (2002). Infliximab retreatment in adults and children with Crohn’s disease: risk factors for the development of delayed severe systemic reaction. Am J Gastroenterol.

[131] Bianchi A, Valluzzi R, Crisafulli G, Bottau P, Caimmi S, Franceschini F, Liotti L, Mori F, Riscassi S, Saretta F, Scavone S, Caffarelli C (2024). Drug-induced anaphylaxis in children Biomedicines.

[132] Blumenthal KG (2018). The role of the clinical history in drug allergy prediction. J Allergy Clin Immunol Pract.

[133] Exius R, Gabrielli S, Abrams EM, O’Keefe A, Protudjer JLP, Lavine E (2021). Establishing amoxicillin allergy in children through direct graded oral challenge (GOC): Evaluating risk factors for positive challenges, safety, and risk of cross-reactivity to cephalosporines. J Allergy Clin Immunol Pract.

[134] Kowalic A, de Monestrol I, Sorjonen K, Brockow K, Gulen T (2022). Antibiotic hypersensitivity in cystic fibrosis- Low frequency of anaphylaxis over 16000 courses. Br J Clin Pharmacol.

